# Functional outcomes and quality of life after endovascular thrombectomy in large and extensive infarcts (ASPECTS 0–5): a multicentre registry study

**DOI:** 10.1093/esj/aakag131

**Published:** 2026-11-02

**Authors:** Hannah Asperger, Felix J Bode, Taraneh Ebrahimi, Lara Klehr, Julia Layer, Julius Meißner, Louisa Nitsch, Omid Shirvani, Christian S Thielscher, Niklas M von Danwitz, Svenja Odensaß, Sebastian Stösser, Franziska Dorn, Gabor C Petzold, Johannes Weller, the GSR-ET Investigators

**Affiliations:** Department of Vascular Neurology, University Hospital Bonn, Bonn, Germany; German Center for Neurodegenerative Diseases (DZNE) Bonn, Bonn, Germany; Department of Vascular Neurology, University Hospital Bonn, Bonn, Germany; Department of Vascular Neurology, University Hospital Bonn, Bonn, Germany; German Center for Neurodegenerative Diseases (DZNE) Bonn, Bonn, Germany; Department of Vascular Neurology, University Hospital Bonn, Bonn, Germany; Department of Vascular Neurology, University Hospital Bonn, Bonn, Germany; German Center for Neurodegenerative Diseases (DZNE) Bonn, Bonn, Germany; Department of Vascular Neurology, University Hospital Bonn, Bonn, Germany; Department of Vascular Neurology, University Hospital Bonn, Bonn, Germany; Department of Neuroimmunology, University Hospital Bonn, Bonn, Germany; Department of Vascular Neurology, University Hospital Bonn, Bonn, Germany; German Center for Neurodegenerative Diseases (DZNE) Bonn, Bonn, Germany; Department of Vascular Neurology, University Hospital Bonn, Bonn, Germany; German Center for Neurodegenerative Diseases (DZNE) Bonn, Bonn, Germany; Department of Vascular Neurology, University Hospital Bonn, Bonn, Germany; German Center for Neurodegenerative Diseases (DZNE) Bonn, Bonn, Germany; Department of Neuroradiology, University Hospital Bonn, Bonn, Germany; Department of Vascular Neurology, University Hospital Bonn, Bonn, Germany; Department of Neuroradiology, University Hospital Bonn, Bonn, Germany; Department of Vascular Neurology, University Hospital Bonn, Bonn, Germany; German Center for Neurodegenerative Diseases (DZNE) Bonn, Bonn, Germany; Department of Vascular Neurology, University Hospital Bonn, Bonn, Germany; Department of Neuroimmunology, University Hospital Bonn, Bonn, Germany; Department of Neurooncology, University Hospital Bonn, Bonn, Germany

**Keywords:** cerebral infarction, endovascular procedures, patient selection, quality of life, registries, thrombectomy, tomography, treatment outcome, X-ray computed

## Abstract

**Introduction:**

Endovascular thrombectomy (EVT) is established for large infarcts, but remains cautiously applied in extensive infarcts with an Alberta Stroke Program Early CT Score (ASPECTS) of 0–2. Real-life outcome data remain limited.

**Patients and methods:**

Retrospective analysis of the German Stroke Registry-Endovascular Treatment comparing extensive (ASPECTS 0–2) and large infarcts (ASPECTS 3–5) in patients with anterior-circulation occlusion and EVT. The primary outcome was the ordinal 90-day mRS distribution; secondary outcomes were mRS 0–3, survival, quality of life (3-level European Quality of Life-5 Dimensions) and living condition.

**Results:**

Of 18,069 screened patients, 889 were analysed (ASPECTS 1–2, *n* = 89; ASPECTS 3–5, *n* = 800); none had ASPECTS 0. With ASPECTS 1–2, 20.2% achieved mRS 0–3 and 55.1% died within 90 days. In the model including successful reperfusion, no statistically significant association was detected between infarct group and the 90-day mRS distribution (common odds ratio [cOR] 0.80; 95% CI, 0.53–1.23) or mRS 0–3 (adjusted odds ratio [aOR] 0.89; 95% CI, 0.64–1.25), whereas successful reperfusion was associated with both (cOR 3.68; 95% CI, 2.31–5.85 and aOR 4.10; 95% CI, 2.45–6.86). Among survivors, median European Quality of Life-5 Dimensions utility index was 0.445 vs 0.541 (*n* = 27/324), and 51.5% vs 50.9% lived at home (*n* = 33/411).

**Discussion:**

An extensive infarct carried a challenging prognosis, yet acceptable outcome and non-institutional living remained achievable in a relevant minority. An independent association of infarct group with outcome was not detected.

**Conclusion:**

An acceptable outcome remained attainable in selected patients with extensive infarction, particularly after successful reperfusion. Lacking a medically managed comparator, these data describe prognosis rather than treatment effect.

## Graphical Abstract

**Graphical Abstract:**
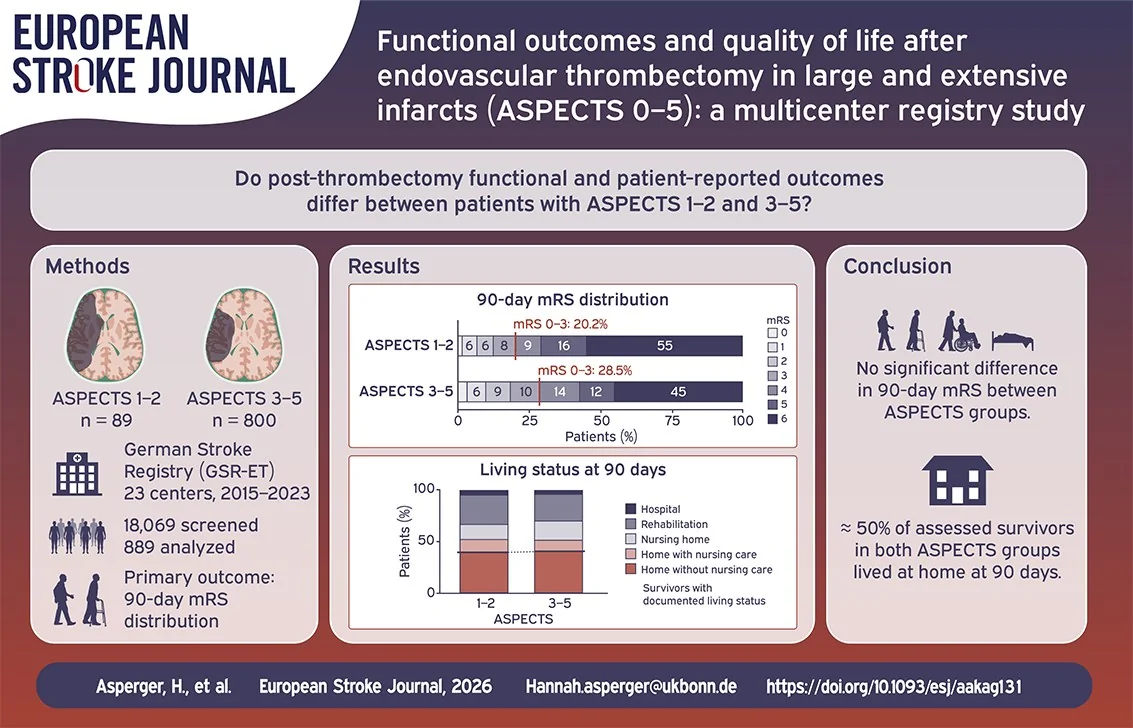

## Introduction

Following the pivotal trials in 2015, EVT has been established as the standard of care in acute ischaemic stroke with large-vessel occlusion, with intravenous thrombolysis (IVT) added in eligible patients.^1^^,^^2^ Patient selection has historically relied on clinical severity, time from onset, vessel status and early ischaemic changes on baseline imaging. The Alberta Stroke Program Early CT Score (ASPECTS) is a 10-point rating scale for early ischaemic changes on non-contrast CT, ranging from 10, indicating no early ischaemic changes, to 0, indicating ischaemic changes in all predefined regions.^3^

In recent years, the indication for EVT has expanded towards later treatment windows and larger ischaemic cores.^4–9^ However, many large-infarct trials focused primarily on ASPECTS 3–5, while patients with ASPECTS 0–2 were excluded, underrepresented or analysed only in small subgroups. The LASTE trial, which included selected patients with ASPECTS 0–2, and meta-analytic evidence indicate that EVT also benefits patients with very low ASPECTS.^10^^,^^11^

Despite these data, real-world evidence regarding EVT in extensive infarct remains limited. Patients with ASPECTS 0–2 are less frequently treated with EVT in clinical practice, and observational studies often report aggregated low-ASPECTS cohorts that also include ASPECTS 3–5.^12^ Additional data on procedural safety, reperfusion, functional outcome, mortality and post-acute living situation are needed to support clinical decision-making and to assess the generalisability of trial findings.

We therefore analysed patients from the German Stroke Registry-Endovascular Treatment (GSR-ET) who underwent EVT for anterior-circulation large-vessel occlusion with baseline ASPECTS 0–5. We compared selected thrombectomy-treated patients with established extensive infarct, defined as ASPECTS 0–2, with patients with ASPECTS 3–5 and assessed procedural and clinical outcomes after EVT. Baseline ASPECTS is available before the decision to treat, whereas the reperfusion grade is known only after the procedure. We therefore examined both as prognostic factors acting at different stages of the treatment pathway rather than as competing predictors. The primary research question was whether baseline infarct extent (ASPECTS 0–2 vs 3–5) is independently associated with the 90-day distribution of the mRS in selected patients undergoing EVT for anterior-circulation large-vessel occlusion with baseline ASPECTS 0–5, and how outcome related to the reperfusion status attained during the procedure.

## Patients and methods

### Study design

This retrospective cohort study enrolled patients from the GSR-ET (NCT03356392). The GSR-ET is an ongoing, academic, open-label, multicentre registry of patients with acute ischaemic stroke treated with EVT in Germany. Patients were prospectively recruited between May 2015 and December 2023 at comprehensive stroke centres in Germany. The full details of the registry have been published previously.^13^^,^^14^ The GSR-ET was approved by the ethics committee of Ludwig Maximilian University Munich (approval number 689-15) and by the local ethics committees of the participating centres. The study followed the Strengthening the Reporting of Observational Studies in Epidemiology (STROBE) guidelines for cohort studies.

### Study cohort and assessments

For the present analysis, adult patients receiving EVT for acute ischaemic stroke due to occlusion of the anterior circulation and a baseline ASPECTS of 0–5 were considered. Anterior-circulation occlusion was defined as occlusion of the internal carotid artery (ICA; extracranial or intracranial segment), the M1 segment or the M2 segment of the MCA. Established extensive infarct was defined as an ASPECTS of 0–2, and established large infarct was defined as an ASPECTS of 3–5. No patient with a recorded baseline ASPECTS of 0 was identified in the available registry data. The extensive-infarct group is therefore referred to as ASPECTS 1–2 wherever the observed study population is described, while ASPECTS 0–2 denotes the eligibility criterion. Patients were required to have 90-day mRS data available after carrying forward in-hospital death as 90-day mRS 6.

Stroke severity was assessed using the NIHSS, and outcome and premorbid disability were rated using the mRS. Baseline imaging, digital subtraction angiograms and follow-up imaging were assessed by local investigators. The baseline ASPECTS on pre-treatment imaging was used to assess the extent of early ischaemic changes. The modality used for baseline ASPECTS assessment was recorded as non-contrast CT, CT perfusion or MRI. For patients recorded as CT perfusion, the registry does not establish whether ASPECTS was scored on non-contrast CT images or on perfusion maps. Reperfusion success was defined as a modified thrombolysis in cerebral infarction (mTICI) score of 2b–3 according to local assessment.^15^ Symptomatic ICH was operationalised using an ECASS II-based definition: reported haemorrhage on 24-h follow-up imaging with an increase of at least 4 NIHSS points from admission.^16^ The registry did not record haemorrhage subtype or adjudicate whether haemorrhage caused neurological deterioration. When haemorrhage was reported but either the admission or 24-h NIHSS was missing, sICH status could not be determined and was treated as missing. Procedural and in-hospital complications were recorded by local investigators. Periprocedural complications comprised vessel dissection or perforation, distal clot migration or embolisation, vasospasm and device malfunction. In-hospital complications up to discharge comprised intracranial haemorrhage, sICH, malignant MCA infarction, recurrent ischaemic stroke, myocardial infarction and groin-site haematoma or pseudoaneurysm.

Health-related quality of life (HRQoL) was assessed at 90-day follow-up using the 3-level European Quality of Life-5 Dimensions (EQ-5D-3L) questionnaire. The EQ-5D utility index for each individual health state was calculated using the UK population value set.^17^ On this index, 1 represents optimal health; quality-of-life analyses were restricted to surviving patients.

### Outcomes

The primary outcome was the distribution of the mRS across its full range at 90-day follow-up, analysed as an ordinal shift. Secondary outcomes were acceptable functional outcome, defined as mRS 0–3 and survival at 90-day follow-up. Further secondary outcomes were excellent outcome (mRS 0–1), good functional outcome (mRS 0–2), sICH, procedural and in-hospital complications, 24-h NIHSS, discharge mRS, HRQoL and living condition at 90-day follow-up. Because mRS 0–3 can only indicate an acceptable outcome if it does not represent worsening beyond substantial premorbid disability, analyses using mRS 0–3 were additionally summarised among patients with premorbid mRS ≤ 3.

### Statistical analysis

Continuous variables are summarised as median and IQR, and categorical variables are summarised as counts and percentages. Variables were compared between groups using the Mann–Whitney *U* test, Fisher exact test or chi-square test, where appropriate. Missing values were summarised descriptively for key variables. Multivariable models were fitted using complete cases; no imputation was performed. Admission-to-groin time was included as a covariate to limit exclusions due to missing timing data. In contrast, the interval from symptom onset, or last seen well when onset was unknown, to groin puncture was unavailable in 150 of 889 patients. Missingness was higher with CT perfusion- or MRI-based ASPECTS assessment than with non-contrast CT (31.1%, 20.4% and 15.7%, respectively). This interval was examined in a sensitivity analysis.

The primary analysis used proportional-odds ordinal logistic regression of the full 90-day mRS distribution. Common odds ratios (cORs) were expressed for a shift towards lower mRS scores. Logistic regression was used for the secondary outcomes mRS 0–3 and survival, with results reported as adjusted odds ratios (aORs).

**Figure 1:**
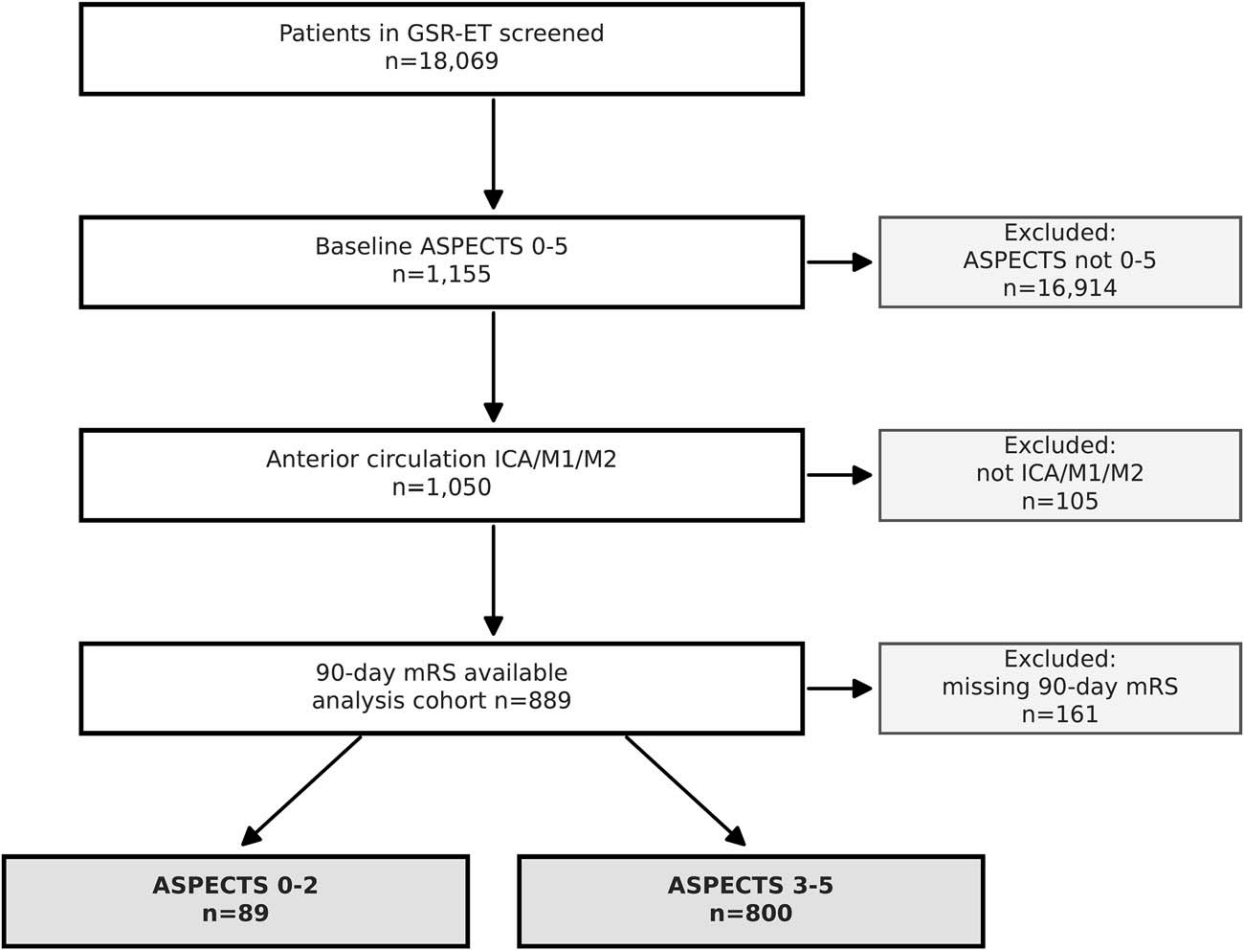
Cohort flow. Patients from the GSR-ET were screened for baseline ASPECTS 0–5, anterior-circulation ICA/M1/M2 occlusion, EVT and available 90-day mRS after carrying forward in-hospital death. ASPECTS 0–2 was defined as extensive infarct and ASPECTS 3–5 as large infarct. No patient with an ASPECTS of 0 was included, so the extensive-infarct group comprises patients with ASPECTS 1–2. Abbreviations: ASPECTS = Alberta Stroke Program Early CT Score; GSR-ET = German Stroke Registry-Endovascular Treatment; ICA = internal carotid artery.

Three sequential models were fitted. Model 1 included infarct group, age, sex, admission NIHSS, premorbid mRS, occlusion site, imaging modality, admission-to-groin time and IVT. Model 2 additionally included reperfusion success. Model 3 added the 24-h NIHSS as a post-treatment prognostic marker. Because reperfusion success and the number of thrombectomy manoeuvres are both procedural variables that may lie on the same causal pathway to outcome, the number of manoeuvres was not entered in the sequential models and was added to model 2 in a sensitivity analysis only. Because complete-case sample sizes differed between models, changes in estimates and fit statistics cannot be attributed solely to the added covariates.

The proportional-odds assumption was explored by comparing estimates for infarct group and reperfusion across separate logistic models at each mRS threshold, using the covariates of model 2.

Because CT- and MRI-based scores are not interchangeable, imaging modality was included as a covariate in all adjusted models, sensitivity analyses were restricted to CT-based assessment and to non-contrast CT alone, and the infarct group by modality interaction was tested by a likelihood-ratio test. To test whether an extensive infarct modified the association between successful reperfusion and 90-day functional outcome, additional models including the interaction term were assessed. Model fit was reported using McFadden’s pseudo-*R*^2^ for the ordinal models and Tjur *R*^2^ for the logistic models. Exploratory likelihood-ratio tests compared interaction models fitted to the same patients and did not account for centre clustering. Standard errors in the sequential and sensitivity regression models were clustered on centre; population-averaged models accounting for within-centre correlation and models including calendar year or treatment era (2020–2023 vs 2015–2019) were fitted as sensitivity analyses. All sensitivity analyses used the covariates of model 2 and were performed for the primary ordinal outcome and for mRS 0–3. Collateral status was not recorded in the registry and could not be included. Centre-level treatment patterns were explored descriptively using anonymised centre labels.

Statistical significance was accepted at *P* < .05, and all tests were 2-sided. Due to the exploratory nature of the analysis, no adjustment for multiple comparisons was performed. Statistical calculations were performed using Python and R. Claude Opus 4.8 (Anthropic) was used as a supporting tool for code refinement and language editing; the authors reviewed and take full responsibility for all content of this manuscript.

## Results

### Patient characteristics

Between May 2015 and December 2023, 18,069 GSR-ET patients treated with EVT were screened for eligibility, of whom 889 met the inclusion criteria (Figure 1). Patients with extensive infarct (*n* = 89) had a median age of 77 years (IQR, 68–81) and 57.3% were female. No patient had a baseline ASPECTS of 0; the group comprised 33 patients with an ASPECTS of 1 and 56 with an ASPECTS of 2 and is referred to as ASPECTS 1–2 below. Across the registry, an ASPECTS of 1–2 was recorded in 110 of 14,348 patients with a scored baseline ASPECTS (0.8%) and an ASPECTS of 0 in none. The median admission NIHSS score was 18 (IQR, 15–21), and 40.4% of this group received IVT. Across the whole cohort, 34.8% received IVT, exclusively alteplase; no patient received tenecteplase. The IVT rate declined from 50.0% and 63.6% in 2015 and 2016 to 24.8% and 27.2% in 2022 and 2023. Median onset- or last-seen-well-to-groin time was 6.4 h (IQR, 3.7–11.7) with ASPECTS 1–2 and 4.9 h (IQR, 3.1–10.4) with ASPECTS 3–5, with 50.6% and 40.8% treated beyond 6 h; median admission-to-groin time was 1.2 h in both groups. Successful reperfusion was achieved in 73 of 88 patients with extensive infarct and an available final mTICI grade (83.0%). No statistically significant differences in baseline characteristics were detected between those with extensive infarct and those with large infarct (ASPECTS 3–5, *n* = 800; all *P* > .05; Table 1). Extensive infarct accounted for a median of 6.1% of included patients per centre (range 0%–16.7% across 23 centres; Table S1). Seven of the 23 centres contributed no patient with an ASPECTS of 1–2, and the yearly share varied between 6.5% and 16.7% without a temporal trend (Figure S1). Missingness was low for most key variables and highest for quality-of-life and living-status data (Table S2).

**Table 1:** Baseline, treatment and outcome characteristics by infarct group.

**Characteristic**	**ASPECTS 1–2**	**ASPECTS 3–5**	***P* value**
	**(*n* = 89)**	**(*n* = 800)**	
**Baseline characteristics**			
**Age, years**	77 [68–81]	74 [62–83]	.157
**Female sex**	51/89 (57.3)	403/800 (50.4)	.221
**Premorbid mRS**	0 [0–2]	0 [0–1]	.264
**Admission NIHSS**	18 [15–21]	17 [14–20]	.196
**ASPECTS 1**	33/89 (37.1)	–	–
**ASPECTS 2**	56/89 (62.9)	–	–
**ASPECTS 3**	–	115/800 (14.4)	–
**ASPECTS 4**	–	214/800 (26.8)	–
**ASPECTS 5**	–	471/800 (58.9)	–
**Hypertension**	66/86 (76.7)	575/793 (72.5)	.445
**Diabetes mellitus**	18/84 (21.4)	171/795 (21.5)	1.000
**Dyslipidaemia**	34/83 (41.0)	284/793 (35.8)	.401
**Atrial fibrillation**	34/85 (40.0)	306/791 (38.7)	.816
**NOAC medication**	7/82 (8.5)	50/785 (6.4)	.479
**Phenprocoumon/warfarin**	3/82 (3.7)	31/785 (3.9)	1.000
**Any oral anticoagulation**	10/82 (12.2)	81/785 (10.3)	.571
**Antiplatelet medication**	25/82 (30.5)	221/785 (28.2)	.700
**Treatment and procedural characteristics**			
**IV thrombolysis**	36/89 (40.4)	273/799 (34.2)	.243
**Successful reperfusion, mTICI 2b–3**	73/88 (83.0)	643/790 (81.4)	.885
**Number of manoeuvres**	2 [1–4]	2 [1–3]	.866
**Outcomes**			
**24-h NIHSS**	20 [14–26.25]	17 [12–24]	.055
**Discharge mRS**	5 [5–6]	5 [4–6]	.021
**90-day mRS 0–1**	6/89 (6.7)	77/800 (9.6)	.447
**90-day mRS 0–2**	11/89 (12.4)	147/800 (18.4)	.189
**90-day mRS 0–3**	18/89 (20.2)	228/800 (28.5)	.105
**90-day mortality, mRS 6**	49/89 (55.1)	363/800 (45.4)	.093
**EQ-5D utility index (survivors)^a^**	0.445 [0.345–0.797]	0.541 [0.272–0.802]	.618
**Living at home without nursing care^b^**	13/33 (39.4)	164/411 (39.9)	1.000
**Living at home with nursing care^b^**	4/33 (12.1)	45/411 (10.9)	.774

Abbreviations: ASPECTS, Alberta Stroke Program Early CT Score; EQ-5D, European Quality of Life-5 Dimensions; ICA, internal carotid artery; mTICI, modified thrombolysis in cerebral infarction; NOAC, non-vitamin K antagonist oral anticoagulant.

Values are median [IQR] or *n*/*N* (%). *N* is the number of patients with the variable observed, so denominators differ between rows where data were missing (Table S2). Extensive infarct was defined as ASPECTS 1–2 and large infarct as ASPECTS 3–5. *P* values are from Mann–Whitney *U* tests for continuous variables and Fisher exact or chi-square tests for categorical variables, excluding missing values from the test.

a EQ-5D is restricted to surviving patients with complete EQ-5D utility data (*n* = 27 and *n* = 324).

b Living-status denominators are 33 and 411 survivors with documented 90-day living status, respectively.

### Functional outcome and mortality

At 90-day follow-up, an excellent outcome (mRS 0–1) was achieved in 6.7% of patients with extensive infarct and 9.6% of patients with large infarct, a good functional outcome (mRS 0–2) in 12.4% and 18.4% and an acceptable functional outcome (mRS 0–3) in 20.2% and 28.5%, respectively. Mortality was high in both groups (55.1% vs 45.4%). Functional outcome was better and mortality lower in patients with large infarct, without reaching statistical significance (Table 1). The full 90-day mRS distributions by infarct group are shown in Figure 2A. Symptomatic ICH occurred in 5 of 86 patients with ASPECTS 1–2 (5.8%) and 52 of 787 with ASPECTS 3–5 (6.6%; *P* = 1.000); status was indeterminate in 3 and 13 patients, respectively. Within ASPECTS 1–2, all 5 events occurred after successful reperfusion (5/71 vs 0/15 after unsuccessful reperfusion).

**Figure 2:**
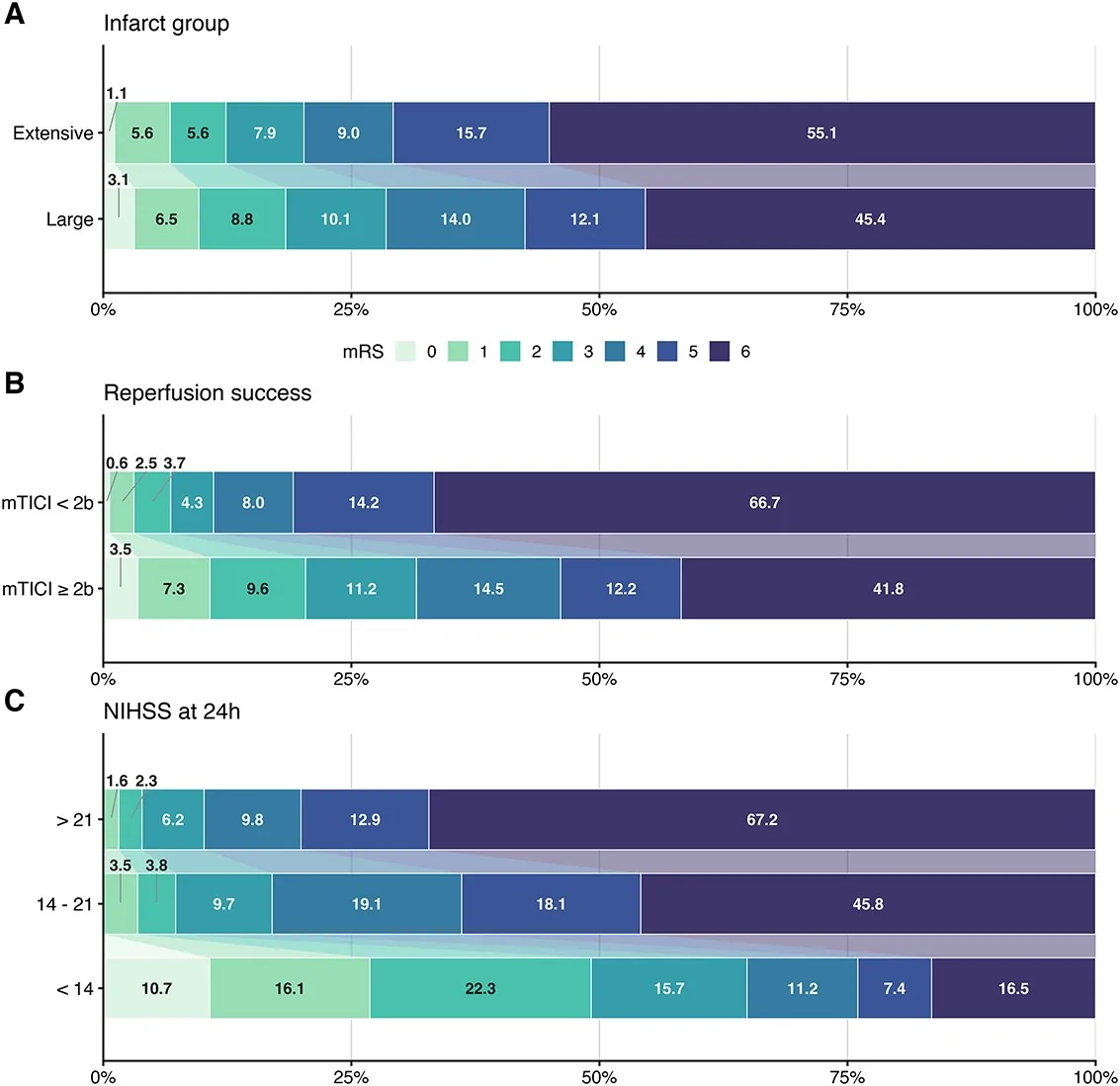
Distribution of 90-day functional outcome (mRS). (A) By infarct group; (B) by reperfusion success; (C) by 24-h NIHSS. Each bar shows the percentage in each mRS category, and connecting bands link the same mRS category between adjacent bars.

Among patients with ASPECTS 3–5, those with sICH less often achieved mRS 0–3 (11.5% vs 30.2%) and had higher mortality (69.2% vs 43.0%) than those without sICH. The 5 events in ASPECTS 1–2 limited interpretation of outcome comparisons. No statistically significant between-group differences in procedural or in-hospital complications were detected (Table S3).

### The role of successful reperfusion

We next assessed how functional outcome related to reperfusion status within each infarct group. Among patients with premorbid mRS ≤ 3 and available final mTICI, acceptable functional outcome (mRS 0–3) after successful reperfusion was achieved in 26.6% of patients with extensive infarct and 34.4% of patients with large infarct, compared with 8.3% and 12.5% after unsuccessful reperfusion, respectively (*P* = .273 for ASPECTS 1–2 and *P* < .001 for ASPECTS 3–5; Figure 3A). In analyses without restriction by premorbid mRS, mRS 0–3 was achieved by 6.7%, 21.1% and 25.7% of patients with ASPECTS 1–2 after mTICI 0–2a, 2b and 3, respectively; corresponding rates in ASPECTS 3–5 were 11.6%, 31.0% and 33.9% (Table S4). In model 2 with separate reperfusion categories, both mTICI 2b (aOR 3.32; 95% CI, 1.94–5.69) and mTICI 3 (aOR 5.15; 95% CI, 2.89–9.19) were associated with mRS 0–3 compared with mTICI 0–2a (Table S4). Higher 24-h NIHSS strata were associated with greater disability and mortality at 90 days (Figure 2C). No statistically significant association between infarct group and the ordinal 90-day mRS distribution was detected in models 1–3 (Table 2). In model 2, the cOR for ASPECTS 1–2 vs 3–5 was 0.80 (95% CI, 0.53–1.23), while successful reperfusion was associated with a shift towards lower mRS scores (cOR 3.68; 95% CI, 2.31–5.85).

**Figure 3:**
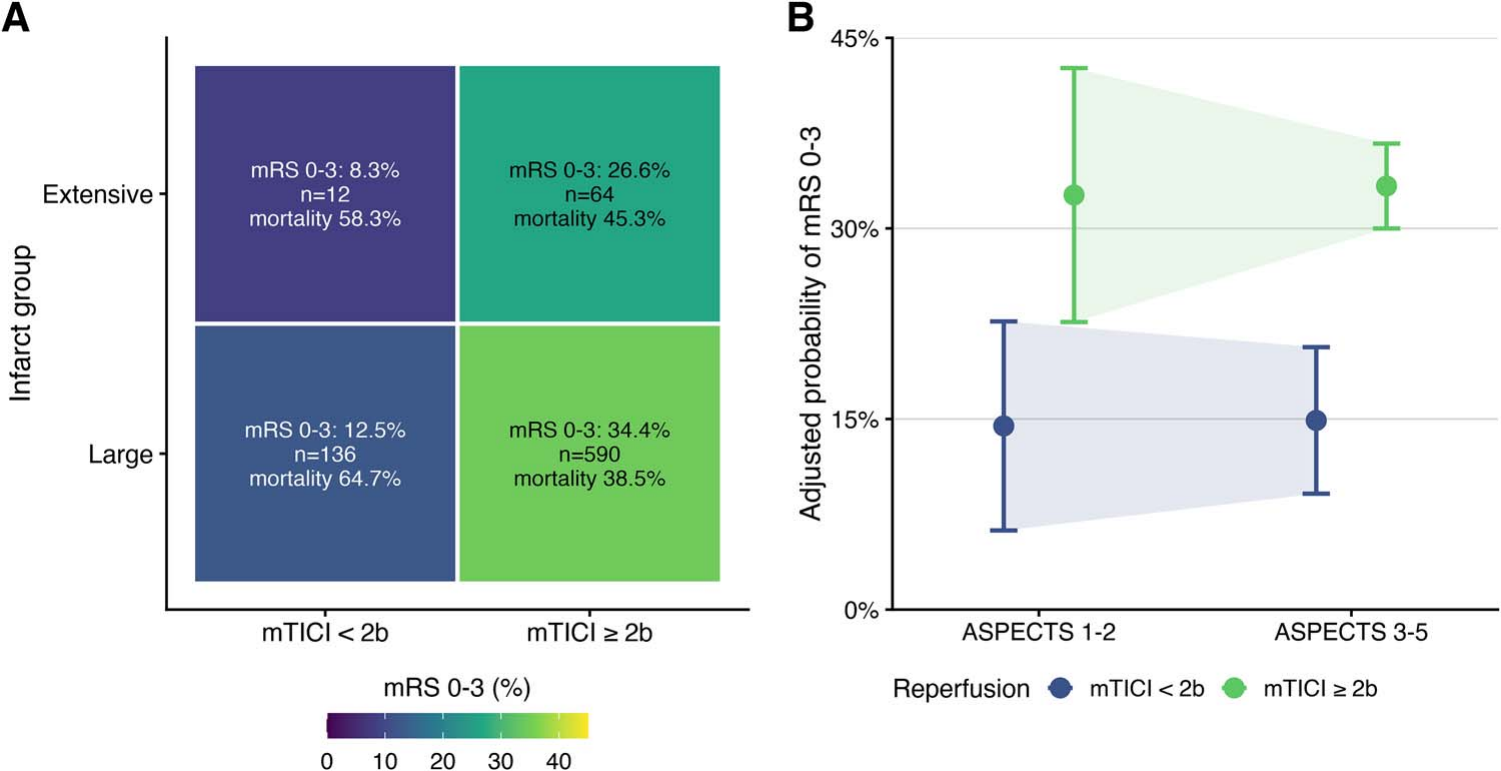
Functional outcome by infarct group and reperfusion success. (A) Proportion of patients with 90-day mRS 0–3 by infarct group and final reperfusion status, restricted to patients with premorbid mRS ≤ 3 and available final mTICI (*n* = 802: 76 with ASPECTS 1–2 and 726 with ASPECTS 3–5). Each cell shows the mRS 0–3 percentage, the cell denominator and 90-day mortality; the panel is descriptive and unadjusted. (B) Adjusted marginal probabilities of mRS 0–3 among patients with premorbid mRS ≤ 3 and complete model covariates (*n* = 755), from a logistic regression model with the covariates of model 2 (infarct group, successful reperfusion, age, sex, admission NIHSS, premorbid mRS, occlusion site, imaging modality, admission-to-groin time and IV thrombolysis). Error bars indicate 95% CIs calculated by the delta method using conventional, non-clustered standard errors. Successful reperfusion was defined as final mTICI 2b–3. Abbreviations: ASPECTS = Alberta Stroke Program Early CT Score; mTICI, modified thrombolysis in cerebral infarction.

**Table 2:** Sequential ordinal analysis of the 90-day mRS (primary outcome).

**Predictor**	**Model 1 initial**	**Model 2 + reperfusion**	**Model 3 + 24-h NIHSS**
**Common odds ratio (95% CI) for a shift towards a better (lower) 90-day mRS**			
**ASPECTS 1–2 vs 3–5**	0.79 (0.57–1.10) *P* = .164	0.80 (0.53–1.23) *P* = .311	1.02 (0.63–1.64) *P* = .951
**Age, per year**	0.95 (0.94–0.96) *P* < .001	0.94 (0.93–0.96) *P* < .001	0.93 (0.92–0.94) *P* < .001
**Female sex**	0.83 (0.59–1.16) *P* = .280	0.84 (0.60–1.16) *P* = .282	0.86 (0.58–1.28) *P* = .461
**Admission NIHSS, per point**	0.89 (0.87–0.92) *P* < .001	0.89 (0.87–0.92) *P* < .001	0.94 (0.92–0.97) *P* < .001
**Premorbid mRS, per point**	0.73 (0.64–0.84) *P* < .001	0.73 (0.64–0.83) *P* < .001	0.75 (0.67–0.86) *P* < .001
**M1 vs ICA occlusion**	1.20 (0.95–1.51) *P* = .121	1.13 (0.90–1.41) *P* = .289	1.05 (0.76–1.43) *P* = .779
**M2 vs ICA occlusion**	2.04 (1.41–2.95) *P* < .001	2.51 (1.83–3.44) *P* < .001	2.10 (1.48–2.99) *P* < .001
**ASPECTS on MRI vs CT**	1.38 (0.84–2.27) *P* = .201	1.40 (0.87–2.24) *P* = .168	1.19 (0.83–1.70) *P* = .344
**Admission-to-groin, per h**	0.91 (0.82–1.01) *P* = .065	0.94 (0.82–1.07) *P* = .363	0.99 (0.91–1.08) *P* = .898
**IV thrombolysis**	1.28 (1.02–1.62) *P* = .037	1.21 (0.96–1.52) *P* = .101	1.32 (0.94–1.85) *P* = .112
**Successful reperfusion**	–	3.68 (2.31–5.85) *P* < .001	2.62 (1.64–4.20) *P* < .001
**24-h NIHSS, per point**	–	–	0.90 (0.85–0.95) *P* < .001
**Observations**	818	810	730
**McFadden pseudo-*R*^2^**	0.128	0.147	0.203

Abbreviations: ASPECTS, Alberta Stroke Program Early CT Score; ICA, internal carotid artery.

Values are common odds ratios (95% CI) and *P* values from proportional-odds ordinal logistic regression across the full 90-day mRS range. A common odds ratio above 1 indicates a shift towards a better (lower) mRS. Model 1 includes infarct group, demographic and clinical characteristics, imaging modality, admission-to-groin time and IV thrombolysis; model 2 adds reperfusion success; model 3 adds the 24-h NIHSS. The number of thrombectomy manoeuvres was not entered in the sequential models and is examined in a sensitivity analysis (Table S9). Standard errors are clustered on centre. Internal carotid artery occlusion is the reference category for occlusion site. The proportional-odds assumption is examined in Table S10; the dichotomous mRS 0–3 and survival analyses are reported in Tables S5 and S6.

In the secondary analyses, successful reperfusion was associated with higher odds of mRS 0–3 and survival, whereas no statistically significant association with infarct group was detected (Tables S5 and S6). In model 3 for mRS 0–3, each additional point on the 24-h NIHSS was associated with lower odds of this outcome (aOR 0.89; 95% CI, 0.84–0.93). No statistically significant interaction between infarct group and reperfusion success was detected for the ordinal mRS (likelihood-ratio test *P* = .576) or for mRS 0–3 (*P* = .947).

The recorded modality for baseline ASPECTS assessment was non-contrast CT in 740 patients, CT perfusion in 45 and MRI in 98; modality was missing or unclassifiable in 6 patients (Table S7). In the sensitivity analysis of the primary ordinal model restricted to CT-based assessment, the cOR for ASPECTS 1–2 vs 3–5 was 0.73 (95% CI, 0.47–1.12); restriction to non-contrast CT yielded a cOR of 0.74 (95% CI, 0.46–1.17). The corresponding aORs for mRS 0–3 were 0.67 (95% CI, 0.44–1.03) and 0.66 (95% CI, 0.43–1.03; Table S8).

In further sensitivity analyses of the primary ordinal model, the cOR for infarct group was 0.83 (95% CI, 0.56–1.25) after adding the number of thrombectomy manoeuvres, 0.80 (95% CI, 0.53–1.21) after adding calendar year, 0.81 (95% CI, 0.53–1.24) after adding treatment era, 0.83 (95% CI, 0.55–1.24) in the population-averaged model and 0.85 (95% CI, 0.52–1.37) when admission-to-groin time was replaced with onset- or last-seen-well-to-groin time (*n* = 703). Successful reperfusion remained associated with a shift towards lower mRS scores in all sensitivity analyses (cOR 3.08–4.10). Neither calendar year (cOR per year 0.95; 95% CI, 0.88–1.02) nor the later treatment era (cOR 0.86; 95% CI, 0.68–1.10) was significantly associated with the ordinal mRS, whereas both were associated with lower odds of mRS 0–3 (aOR per year 0.91; 95% CI, 0.85–0.98; later era, aOR 0.72; 95% CI, 0.54–0.95). The estimates for infarct group were not materially changed in either analysis (Table S9).

### Quality of life and home reintegration

To characterise patient-centred outcomes beyond functional disability, we assessed health-related quality of life and living situation at 90 days. European Quality of Life-5 Dimensions utility data at 90 days were available for 27 of 40 survivors with ASPECTS 1–2 (67.5%) and 324 of 437 survivors with ASPECTS 3–5 (74.1%). Among these patients, the median EQ-5D utility index was 0.445 (IQR 0.345–0.797) vs 0.541 (IQR 0.272–0.802) for extensive and large infarct, respectively (*P* = .618; Figure 4A). Living status was available for 33 of 40 survivors with ASPECTS 1–2 and 411 of 437 survivors with ASPECTS 3–5. Living at home without nursing care was reported for 13/33 (39.4%) and 164/411 (39.9%), and living at home with nursing care for 4/33 (12.1%) and 45/411 (10.9%), respectively (Figure 4B).

**Figure 4:**
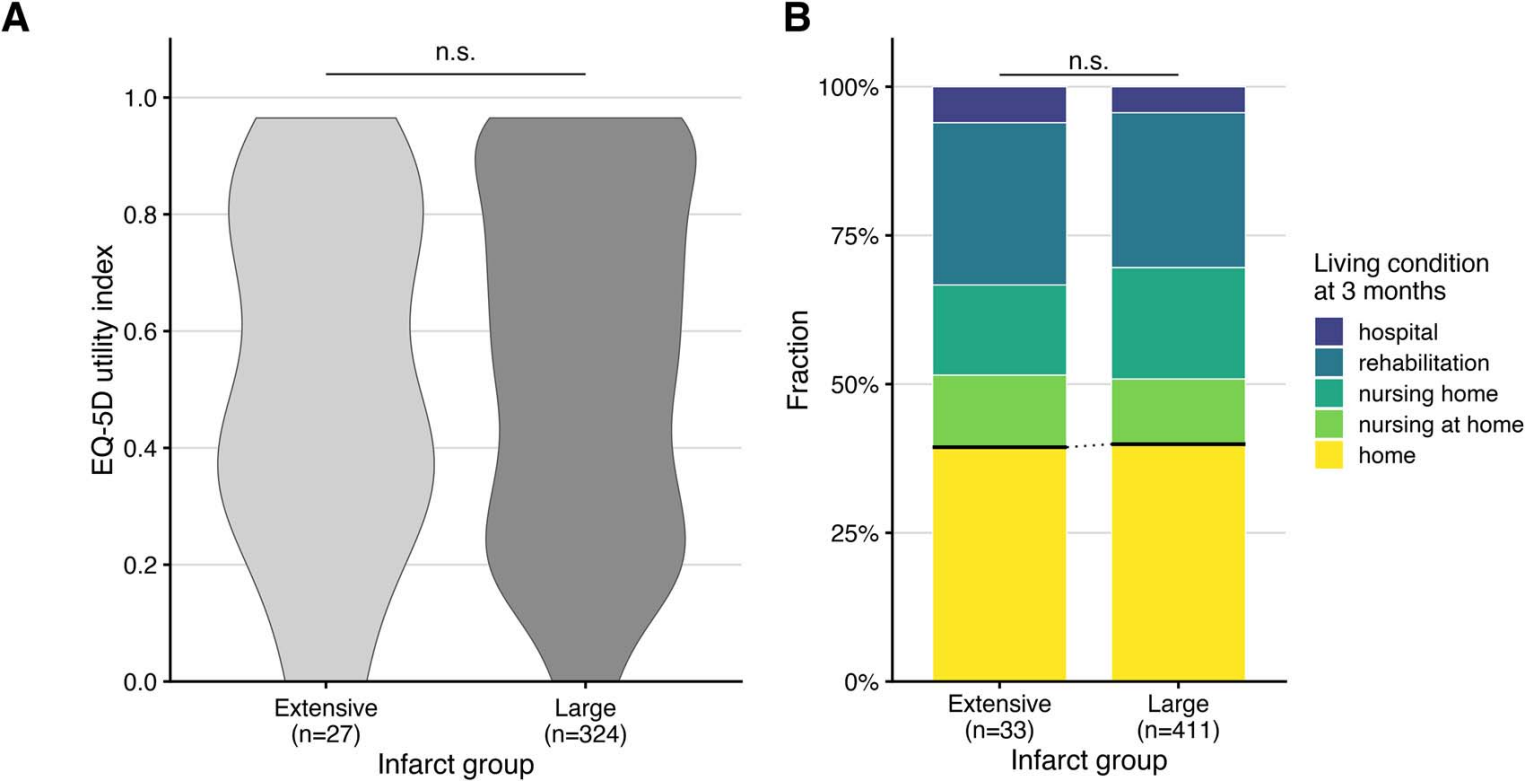
Quality of life and living condition after EVT. (A) EQ-5D utility index at 90 days among surviving patients with an available utility index (*n* = 27 of 40 survivors with ASPECTS 1–2 and *n* = 324 of 437 with ASPECTS 3–5). (B) Living condition at 90 days among surviving patients with documented living status (*n* = 33 of 40 survivors with ASPECTS 1–2 and *n* = 411 of 437 with ASPECTS 3–5). The number of patients with available data is given below each group label. Abbreviations: ASPECTS = Alberta Stroke Program Early CT Score; EQ-5D = European Quality of Life-5 Dimensions.

## Discussion

Our study reports functional outcomes and HRQoL after EVT in selected patients with extensive infarct. Endovascular thrombectomy was infrequently performed in extensive infarct, illustrating the only recently developing evidence for an EVT benefit and the cautious approach taken in clinical practice towards this subgroup, which has been excluded from most previous clinical trials.^11^ No statistically significant association of infarct group with ordinal mRS, mRS 0–3 or survival was detected in the adjusted analyses. Nevertheless, the confidence intervals do not exclude a clinically relevant prognostic contribution of baseline infarct extent. ASPECTS informs assessment before treatment, whereas reperfusion success is known only after the procedure. Their prognostic value therefore cannot be compared on the basis of statistical significance alone. The strong association of the 24-h NIHSS with outcome indicates that post-procedural neurological status carries substantial prognostic information beyond baseline infarct.

These observations are in line with previous observational thrombectomy series in low-ASPECTS stroke. Successful reperfusion has been associated with reduced disability and mortality in patients with a pre-treatment diffusion-weighted imaging ASPECTS (DWI-ASPECTS) ≤ 6,^18^ clinical-practice analyses of large baseline infarcts have identified reperfusion and clinical severity rather than the ASPECTS category alone as key determinants of outcome,^19^ and outcomes have been reported to be broadly comparable between ASPECTS 2–3 and 4–5.^20^ Not all data are concordant, however, as some series have suggested limited benefit and an increased haemorrhage risk in the most extensive infarcts.^21^ No statistically significant difference in procedural or in-hospital complications was detected between infarct groups. Symptomatic ICH was associated with worse outcomes in ASPECTS 3–5, while the 5 events in ASPECTS 1–2 limited interpretation. Our sICH definition did not establish whether haemorrhage caused neurological deterioration.

Health-related quality of life after EVT in low-ASPECTS stroke has been examined previously, with long-term EQ-5D utility reported to depend on reperfusion grade in a single-centre cohort of patients with baseline ASPECTS 0–5.^22^ Among survivors with available data, the median EQ-5D utility index was 0.445 for extensive infarct and 0.541 for large infarct, without a statistically significant difference. Among the 40 survivors with ASPECTS 1–2, living status was available for 33 patients. Of these, 17 (51.5%) were living at home, with or without nursing care, at 90 days. Unadjusted rates of acceptable functional outcome and survival were 8.3 and 9.7 percentage points higher with large than extensive infarct, respectively, whereas no statistically significant difference in home reintegration among survivors was detected. On the other hand, our study indicates that in these selected patients, acceptable outcomes are indeed not rarely achieved in clinical practice.^6–9^

The reported rate of acceptable outcomes of 20.2% is broadly consistent with another study of functional outcomes after EVT in extensive infarct, in which an mRS of 0–3 was reported in 31% of patients.^23^ Differences from our cohort may reflect differences in baseline characteristics and case mix, and the 90-day mortality reported in that study (41.4%) likewise underscores the high mortality of this population, although it was lower than the 55.1% observed here.

Notably, the association between successful reperfusion and outcome in extensive infarct highlights the limitations of ASPECTS: any early infarction of a given region is considered, irrespective of the hypoattenuation degree. To overcome this limitation, it has been shown that the extent of hypoattenuation is predictive of outcomes following reperfusion and predicts reperfusion benefit in low ASPECTS.^24^^,^^25^

Differences in patient characteristics across imaging modalities may have contributed to the observed outcome differences. In the analyses restricted to CT-based assessment, the point estimates for ASPECTS 1–2 vs 3–5 were lower than in the full cohort for both the ordinal mRS and mRS 0–3, with confidence intervals that included 1; these analyses do not exclude a clinically relevant association between lower ASPECTS and poorer outcome. For patients recorded as CT perfusion, the registry does not establish whether ASPECTS was scored on non-contrast CT or on perfusion maps; restriction to non-contrast CT yielded similar estimates.

Several limitations should be considered. This study is observational and therefore subject to treatment-selection bias and residual confounding. All included patients underwent EVT and no medically managed comparison group was available. The analyses therefore describe prognostic associations within a treated cohort and cannot estimate the effect of EVT relative to best medical treatment in extensive infarction; that question is addressed by the randomised large-core trials, which included a medically managed comparator.^11^ Patients with ASPECTS 1–2 who underwent EVT represent a selected subgroup. Treatment decisions may have depended on prognostic factors not fully captured in the registry, limiting generalisability and leaving potential for residual confounding. The findings cannot be extrapolated to patients with ASPECTS 0, who were not represented. ASPECTS and reperfusion grades were assessed locally, without central or blinded adjudication. Inter-rater reliability data were unavailable, and measurement variability may have affected classification around the ASPECTS group boundary. Data on decompressive hemicraniectomy were not systematically available, precluding assessment of its association with outcome. European Quality of Life-5 Dimensions utility data were missing for 13 of 40 survivors with ASPECTS 1–2 (32.5%) and 113 of 437 with ASPECTS 3–5 (25.9%), and living status for 7 (17.5%) and 26 (5.9%), respectively; the quality-of-life and living-status analyses should therefore be interpreted as secondary descriptive findings. The small ASPECTS 1–2 subgroup (*n* = 89) limits the precision of between-group comparisons. Non-significant findings should not be interpreted as evidence of equivalence.

Our findings describe the prognosis of selected patients with extensive infarction treated with EVT in routine practice: an acceptable functional outcome remained attainable in approximately 1 in 5, half of survivors with documented living status were living at home, with or without nursing care, and successful reperfusion was associated with better functional outcome. The 24-h NIHSS was associated with 90-day outcome and may inform subsequent discussions of prognosis with patients and their families. Cost-effectiveness of EVT has been reported in large infarct but was not assessed in the present study.^26^^,^^27^

## Conclusion

Our study contributes important real-life data on the outcomes of selected patients with extensive infarct treated with EVT, highlighting the challenges in achieving acceptable outcomes in this subgroup. Nevertheless, acceptable functional outcomes and living at home were observed in a subset of patients. Successful reperfusion was associated with better functional outcome. Further research is needed to optimise treatment allocation for this severely affected subgroup of patients with large-vessel occlusion stroke in the anterior circulation.

## Supplementary Material

supplements_revised_2_aakag131

## Data Availability

The data that support the findings of this study are not publicly available because of data protection restrictions applying to the German Stroke Registry-Endovascular Treatment. Data are available from the GSR-ET steering committee upon reasonable request and after approval by the participating centres and the responsible ethics committees.

## Trial registration

clinicaltrials.gov NCT03356392.

## GSR-ET collaborators

A. Alegiani, J. Berrouschot, T. Boeck-Behrens, G. Bohner, J. Borggrefe, A. Bormann, M. Braun, F. Dorn, B. Eckert, U. Ernemann, M. Ernst, J. Fiehler, K. Gröschel, J. Hattingen, G. F. Hamann, C. Heitkamp, K. H. Henn, F. Keil, L. Kellert, H. Leischner, A. Ludolph, I. Maier, O. Nikoubashman, C. H. Nolte, M. Petersen, S. Poli, G. C. Petzold, A. Reich, J. Röther, C. Riedel, J. H. Schäfer, M. Schell, P. Schellinger, E. Siebert, F. Stögbauer, G. Thomalla, S. Tiedt, C. Trumm, T. Uphaus, S. Wunderlich.
