# Supplementary material for: Functional outcomes and quality of life after endovascular thrombectomy in large and extensive infarcts (ASPECTS 0–5): a multicentre registry study
Source: Eur Stroke J. 2026 Nov 2;11(11):aakag131. doi: 10.1093/esj/aakag131 (PMC13725919; doi:10.1093/esj/aakag131)
Supplement: Supplementary file 1 [file supplements_revised_2_aakag131.docx]

Supplementary Material

**Functional Outcomes and Quality of Life After Endovascular Thrombectomy in Large and Extensive Infarcts (ASPECTS 0-5): A Multicenter Registry Study**

**Supplementary Table 1. Center-level treatment patterns by center volume.**
Centers are grouped by the number of included patients rather than listed individually to preserve anonymity. Center volume is the number of included patients per center; values are n (%) of patients within each volume stratum. Because several centers contributed few patients, center-level patterns were considered descriptive and were not used for inferential testing. ASPECTS indicates Alberta Stroke Program Early CT Score.

| **Center volume** | **Centers** | **ASPECTS 1-2** | **ASPECTS 3-5** |
| --- | --- | --- | --- |
| ≥50 | 5 | 66 (12.0) | 483 (88.0) |
| 20-49 | 7 | 16 (6.7) | 224 (93.3) |
| 10-19 | 6 | 7 (7.8) | 83 (92.2) |
| <10 | 5 | 0 (0.0) | 10 (100.0) |
| Total | 23 | 89 (10.0) | 800 (90.0) |

**Supplementary Table 2. Missing values for key variables.**
Values are n/N (%) missing. EQ-5D and living-status rows use the 40 and 437 survivors as denominators; all other rows use the full infarct groups (n=89 and n=800). Variables with complete availability in both groups, including age, sex, baseline ASPECTS, 90-day mRS, mRS 0-3, and 90-day mortality, are not shown. The EQ-5D utility index and its individual dimensions were assessed only in surviving patients. ^a^Five records were left blank and one was recorded as not applicable; modality-stratified analyses therefore cover 883 of 889 patients. ASPECTS indicates Alberta Stroke Program Early CT Score; EQ-5D, European Quality of Life-Five Dimensions; mRS, modified Rankin Scale; mTICI, modified Thrombolysis in Cerebral Infarction; NIHSS, National Institutes of Health Stroke Scale.

| **Variable** | **ASPECTS 1-2 missing** | **ASPECTS 3-5 missing** |
| --- | --- | --- |
| Premorbid mRS | 4/89 (4.5) | 18/800 (2.2) |
| Admission NIHSS | 3/89 (3.4) | 10/800 (1.2) |
| ASPECTS imaging modality | 0/89 (0.0) | 6/800 (0.8)^a^ |
| IV thrombolysis | 0/89 (0.0) | 1/800 (0.1) |
| Final mTICI | 1/89 (1.1) | 10/800 (1.2) |
| Successful reperfusion | 1/89 (1.1) | 10/800 (1.2) |
| Number of maneuvers | 8/89 (9.0) | 37/800 (4.6) |
| 24-hour NIHSS | 13/89 (14.6) | 90/800 (11.2) |
| Discharge mRS | 0/89 (0.0) | 22/800 (2.8) |
| EQ-5D utility index (survivors) | 13/40 (32.5) | 113/437 (25.9) |
| 90-day living status (survivors) | 7/40 (17.5) | 26/437 (5.9) |
| EQ-5D mobility (survivors) | 12/40 (30.0) | 100/437 (22.9) |
| EQ-5D self-care (survivors) | 13/40 (32.5) | 99/437 (22.7) |
| EQ-5D usual activities (survivors) | 13/40 (32.5) | 100/437 (22.9) |
| EQ-5D pain/discomfort (survivors) | 13/40 (32.5) | 107/437 (24.5) |
| EQ-5D anxiety/depression (survivors) | 13/40 (32.5) | 109/437 (24.9) |
| Onset-to-admission time | 55/89 (61.8) | 450/800 (56.2) |
| Last-seen-well-to-admission time | 46/89 (51.7) | 474/800 (59.2) |
| Admission-to-groin time | 1/89 (1.1) | 33/800 (4.1) |
| Groin-to-final-reperfusion time | 8/89 (9.0) | 113/800 (14.1) |

**Supplementary Table 3. Periprocedural and in-hospital complications by infarct group.**
Values are n/N (%). Complications were recorded by local investigators as adverse-event items, and an absent entry was treated as no reported event. P values are from Fisher exact tests. Intracranial hemorrhage and symptomatic intracranial hemorrhage were assessed on 24-hour follow-up imaging; symptomatic intracranial hemorrhage additionally required an increase of at least 4 points in the NIHSS. Its denominator is smaller than the group size because sICH status could not be determined in 3 and 13 patients, respectively, in whom a hemorrhage was reported but a NIHSS value was unavailable. ASPECTS indicates Alberta Stroke Program Early CT Score; MCA, middle cerebral artery; NIHSS, National Institutes of Health Stroke Scale.

| **Complication** | **ASPECTS 1-2** | **ASPECTS 3-5** | **p value** |
| --- | --- | --- | --- |
| **Periprocedural** | | | |
| Any periprocedural complication | 16/89 (18.0) | 163/800 (20.4) | 0.677 |
| Vessel dissection or perforation | 1/89 (1.1) | 15/800 (1.9) | 1.000 |
| Distal clot migration or embolization | 5/89 (5.6) | 47/800 (5.9) | 1.000 |
| Vasospasm | 2/89 (2.2) | 39/800 (4.9) | 0.421 |
| Device malfunction | 1/89 (1.1) | 7/800 (0.9) | 0.571 |
| **In-hospital** | | | |
| Any in-hospital complication | 50/89 (56.2) | 434/800 (54.2) | 0.738 |
| Intracranial hemorrhage (24-hour imaging) | 14/89 (15.7) | 130/800 (16.2) | 1.000 |
| Symptomatic intracranial hemorrhage | 5/86 (5.8) | 52/787 (6.6) | 1.000 |
| Malignant MCA infarction | 6/89 (6.7) | 48/800 (6.0) | 0.814 |
| Recurrent ischemic stroke | 3/89 (3.4) | 16/800 (2.0) | 0.426 |
| Myocardial infarction | 0/89 (0.0) | 8/800 (1.0) | 1.000 |
| Groin hematoma | 0/89 (0.0) | 6/800 (0.8) | 1.000 |
| Groin pseudoaneurysm | 0/89 (0.0) | 1/800 (0.1) | 1.000 |

**Supplementary Table 4. Ninety-day outcome by final mTICI grade.**
Descriptive rows are unadjusted and are not restricted by premorbid mRS; percentages use the patients with an available final mTICI grade as denominators (11 of 889 patients had no recorded grade). The registry does not contain an mTICI 2c category, so near-complete reperfusion cannot be separated from complete reperfusion. The adjusted estimates come from Model 2 (Table 2 and Supplementary Table 5) with reperfusion entered as three categories instead of the dichotomy, with standard errors clustered on center; mTICI 0-2a is the reference category. Common odds ratios are expressed for a shift towards lower mRS scores. aOR indicates adjusted odds ratio; ASPECTS, Alberta Stroke Program Early CT Score; CI, confidence interval; cOR, common odds ratio; mRS, modified Rankin Scale; mTICI, modified Thrombolysis in Cerebral Infarction.

| **Final mTICI** | **Infarct** | **n** | **mRS 0-3** | **Mortality** | **Median** |
| --- | --- | --- | --- | --- | --- |
|  | **group** |  | **%** | **%** | **90-day mRS** |
| mTICI 0-2a | ASPECTS 1-2 | 15 | 6.7 | 66.7 | 6 |
| mTICI 0-2a | ASPECTS 3-5 | 147 | 11.6 | 66.7 | 6 |
| mTICI 2b | ASPECTS 1-2 | 38 | 21.1 | 52.6 | 6 |
| mTICI 2b | ASPECTS 3-5 | 310 | 31.0 | 41.6 | 5 |
| mTICI 3 | ASPECTS 1-2 | 35 | 25.7 | 51.4 | 6 |
| mTICI 3 | ASPECTS 3-5 | 333 | 33.9 | 39.6 | 5 |
| **Model 2 with separate reperfusion categories (n=810)** | | | | | |
| **Comparison** | **Ordinal mRS, cOR (95% CI)** | | **mRS 0-3, aOR (95% CI)** | | |
| mTICI 2b vs. 0-2a | 2.91 (1.75-4.86), p<0.001 | | 3.32 (1.94-5.69), p<0.001 | | |
| mTICI 3 vs. 0-2a | 4.69 (2.91-7.55), p<0.001 | | 5.15 (2.89-9.19), p<0.001 | | |

**Supplementary Table 5. Sequential logistic regression models for acceptable 90-day outcome (mRS 0-3), a secondary outcome.**
Values are adjusted odds ratios (95% CI) and p values. Acceptable outcome was defined as 90-day mRS 0-3. Model 1 includes infarct group, demographic and clinical characteristics, imaging modality, admission-to-groin time, and IV thrombolysis; Model 2 adds reperfusion success; Model 3 adds the 24-hour NIHSS. The number of thrombectomy maneuvers was not entered in the sequential models and is examined in a sensitivity analysis (Supplementary Table 9). Standard errors are clustered on center. Internal carotid artery occlusion is the reference category for occlusion site. ASPECTS indicates Alberta Stroke Program Early CT Score; CI, confidence interval; ICA, internal carotid artery; mRS, modified Rankin Scale; MRI, magnetic resonance imaging; NIHSS, National Institutes of Health Stroke Scale.

| **Predictor** | **Model 1 initial** | **Model 2 + reperfusion** | **Model 3 + 24-h NIHSS** |
| --- | --- | --- | --- |
| ASPECTS 1-2 vs. 3-5 | 0.91 (0.64-1.28) p=0.582 | 0.89 (0.64-1.25) p=0.511 | 1.10 (0.77-1.58) p=0.597 |
| Age, per year | 0.95 (0.93-0.96) p<0.001 | 0.95 (0.93-0.96) p<0.001 | 0.93 (0.92-0.95) p<0.001 |
| Female sex | 0.81 (0.51-1.27) p=0.355 | 0.82 (0.52-1.28) p=0.388 | 0.79 (0.43-1.44) p=0.438 |
| Admission NIHSS, per point | 0.89 (0.86-0.92) p<0.001 | 0.89 (0.86-0.92) p<0.001 | 0.95 (0.93-0.97) p<0.001 |
| Premorbid mRS, per point | 0.70 (0.56-0.87) p=0.002 | 0.71 (0.58-0.87) p<0.001 | 0.73 (0.57-0.92) p=0.008 |
| M1 vs. ICA occlusion | 1.08 (0.79-1.46) p=0.640 | 1.04 (0.76-1.41) p=0.812 | 1.01 (0.68-1.51) p=0.964 |
| M2 vs. ICA occlusion | 1.98 (1.20-3.28) p=0.008 | 2.29 (1.31-3.98) p=0.003 | 1.95 (0.95-3.98) p=0.067 |
| ASPECTS on MRI vs. CT | 1.44 (1.00-2.08) p=0.052 | 1.47 (1.02-2.12) p=0.037 | 1.17 (0.80-1.70) p=0.424 |
| Admission-to-groin, per h | 0.89 (0.80-1.00) p=0.045 | 0.92 (0.81-1.04) p=0.185 | 0.99 (0.89-1.10) p=0.892 |
| IV thrombolysis | 1.25 (0.95-1.65) p=0.118 | 1.16 (0.88-1.53) p=0.287 | 1.28 (0.82-2.01) p=0.277 |
| Successful reperfusion | - | 4.10 (2.45-6.86) p<0.001 | 2.98 (1.96-4.55) p<0.001 |
| 24-hour NIHSS, per point | - | - | 0.89 (0.84-0.93) p<0.001 |
| Observations | 818 | 810 | 730 |
| Tjur R^2^ | 0.264 | 0.296 | 0.430 |

**Supplementary Table 6. Sequential logistic regression models for 90-day survival, a secondary outcome.**
Values are adjusted odds ratios (95% CI) and p values. Survival was defined as absence of 90-day mRS 6. Model 1 includes infarct group, demographic and clinical characteristics, imaging modality, admission-to-groin time, and IV thrombolysis; Model 2 adds reperfusion success; Model 3 adds the 24-hour NIHSS. The number of thrombectomy maneuvers was not entered in the sequential models and is examined in a sensitivity analysis (Supplementary Table 9). Standard errors are clustered on center. Internal carotid artery occlusion is the reference category for occlusion site. ASPECTS indicates Alberta Stroke Program Early CT Score; CI, confidence interval; ICA, internal carotid artery; mRS, modified Rankin Scale; MRI, magnetic resonance imaging; NIHSS, National Institutes of Health Stroke Scale.

| **Predictor** | **Model 1 initial** | **Model 2 + reperfusion** | **Model 3 + 24-h NIHSS** |
| --- | --- | --- | --- |
| ASPECTS 1-2 vs. 3-5 | 0.87 (0.54-1.39) p=0.557 | 0.86 (0.49-1.50) p=0.596 | 1.02 (0.57-1.83) p=0.936 |
| Age, per year | 0.94 (0.93-0.96) p<0.001 | 0.94 (0.93-0.95) p<0.001 | 0.92 (0.91-0.94) p<0.001 |
| Female sex | 0.85 (0.59-1.22) p=0.372 | 0.84 (0.58-1.23) p=0.376 | 0.94 (0.63-1.40) p=0.757 |
| Admission NIHSS, per point | 0.92 (0.90-0.94) p<0.001 | 0.92 (0.90-0.94) p<0.001 | 0.96 (0.94-0.99) p=0.003 |
| Premorbid mRS, per point | 0.77 (0.67-0.90) p<0.001 | 0.77 (0.67-0.89) p<0.001 | 0.82 (0.73-0.93) p=0.001 |
| M1 vs. ICA occlusion | 1.18 (0.88-1.57) p=0.270 | 1.11 (0.83-1.50) p=0.480 | 1.06 (0.74-1.52) p=0.748 |
| M2 vs. ICA occlusion | 1.64 (0.89-3.05) p=0.116 | 1.95 (1.13-3.34) p=0.016 | 1.96 (0.99-3.88) p=0.055 |
| ASPECTS on MRI vs. CT | 1.46 (0.69-3.12) p=0.324 | 1.46 (0.69-3.10) p=0.319 | 1.30 (0.73-2.34) p=0.375 |
| Admission-to-groin, per h | 0.93 (0.83-1.05) p=0.268 | 0.96 (0.83-1.11) p=0.566 | 1.00 (0.89-1.11) p=0.955 |
| IV thrombolysis | 1.10 (0.84-1.43) p=0.497 | 1.04 (0.74-1.48) p=0.814 | 1.05 (0.63-1.73) p=0.863 |
| Successful reperfusion | - | 3.32 (1.98-5.58) p<0.001 | 2.55 (1.42-4.58) p=0.002 |
| 24-hour NIHSS, per point | - | - | 0.92 (0.87-0.97) p=0.003 |
| Observations | 818 | 810 | 730 |
| Tjur R^2^ | 0.241 | 0.275 | 0.348 |

**Supplementary Table 7. Baseline characteristics and outcomes by the modality on which the baseline ASPECTS was scored.**
Age and NIHSS are medians. Reperfusion denotes a final mTICI of 2b-3. Modality categories reflect the registry entries. Six patients with missing or unclassifiable modality are not shown. Differences in age, admission NIHSS, and reperfusion rates suggest differences in patient selection across imaging modalities. ASPECTS indicates Alberta Stroke Program Early CT Score; CT, computed tomography; mRS, modified Rankin Scale; mTICI, modified Thrombolysis in Cerebral Infarction; MRI, magnetic resonance imaging; NIHSS, National Institutes of Health Stroke Scale.

| **ASPECTS** | **Infarct** | **n** | **Age** | **NIHSS** | **Reperf.** | **mRS 0-3** | **Mortality** |
| --- | --- | --- | --- | --- | --- | --- | --- |
| **modality** | **group** |  | **median** | **median** | **%** | **%** | **%** |
| Non-contrast CT | ASPECTS 1-2 | 71 | 78 | 18 | 81.4 | 15.5 | 59.2 |
| Non-contrast CT | ASPECTS 3-5 | 669 | 74 | 17 | 81.9 | 27.8 | 47.1 |
| CT perfusion | ASPECTS 1-2 | 5 | 72 | 17 | 60.0 | 0.0 | 80.0 |
| CT perfusion | ASPECTS 3-5 | 40 | 74 | 17 | 80.0 | 15.0 | 45.0 |
| MRI | ASPECTS 1-2 | 13 | 65 | 16 | 100.0 | 53.8 | 23.1 |
| MRI | ASPECTS 3-5 | 85 | 69 | 15 | 80.0 | 42.4 | 29.4 |

**Supplementary Table 8. Sensitivity of Model 2 to the ASPECTS imaging modality, primary ordinal outcome and mRS 0-3.**
All models use the covariates of Model 2 (infarct group, age, sex, admission NIHSS, premorbid mRS, occlusion site, imaging modality, admission-to-groin time, IV thrombolysis, reperfusion success) with standard errors clustered on center; the restricted analyses contain no modality covariate. Common odds ratios are expressed for a shift towards lower mRS scores. CT-based assessment comprises patients recorded as non-contrast CT or CT perfusion; for patients recorded as CT perfusion, the registry does not establish whether ASPECTS was scored on non-contrast CT or on perfusion maps. Exploratory likelihood-ratio tests of the infarct group by imaging modality interaction yielded chi-square 1.233 (1 df, p=0.267) for the ordinal mRS and chi-square 2.709 (1 df, p=0.100) for mRS 0-3; these tests did not account for center clustering. These results do not establish that pooling CT- and MRI-based scores is free from bias. An MRI-only model was not fitted because only 13 patients with ASPECTS 1-2 underwent MRI. aOR indicates adjusted odds ratio; ASPECTS, Alberta Stroke Program Early CT Score; CI, confidence interval; cOR, common odds ratio; CT, computed tomography; MRI, magnetic resonance imaging; mRS, modified Rankin Scale; NIHSS, National Institutes of Health Stroke Scale.

| **Analysis** | **Predictor** | **Ordinal mRS, cOR (95% CI)** | **p value** | **mRS 0-3, aOR (95% CI)** | **p value** |
| --- | --- | --- | --- | --- | --- |
| All patients, modality adjusted (n=810) | ASPECTS 1-2 vs. 3-5 | 0.80 (0.53-1.23) | 0.311 | 0.89 (0.64-1.25) | 0.511 |
| All patients, modality adjusted (n=810) | Successful reperfusion | 3.68 (2.31-5.85) | <0.001 | 4.10 (2.45-6.86) | <0.001 |
| All patients, modality adjusted (n=810) | MRI-based ASPECTS | 1.40 (0.87-2.24) | 0.168 | 1.47 (1.02-2.12) | 0.037 |
| CT-based only (n=719) | ASPECTS 1-2 vs. 3-5 | 0.73 (0.47-1.12) | 0.146 | 0.67 (0.44-1.03) | 0.071 |
| CT-based only (n=719) | Successful reperfusion | 3.26 (2.07-5.13) | <0.001 | 3.81 (2.16-6.73) | <0.001 |
| Non-contrast CT only (n=676) | ASPECTS 1-2 vs. 3-5 | 0.74 (0.46-1.17) | 0.200 | 0.66 (0.43-1.03) | 0.066 |
| Non-contrast CT only (n=676) | Successful reperfusion | 3.08 (1.95-4.86) | <0.001 | 3.58 (2.03-6.32) | <0.001 |

**Supplementary Table 9. Further sensitivity analyses of Model 2, primary ordinal outcome and mRS 0-3.**
Each analysis modifies Model 2 (Table 2 and Supplementary Table 5) as stated. The number of thrombectomy maneuvers, calendar year of admission, and treatment era were each added to Model 2; the number of maneuvers was not part of the sequential models because it may lie on the same causal pathway as reperfusion success. The population-averaged models are generalized estimating equations accounting for within-center correlation. In the timing analysis, admission-to-groin time was replaced with onset- or last-seen-well-to-groin time. Standard errors in all other analyses are clustered on center. Common odds ratios are expressed for a shift towards lower mRS scores. aOR indicates adjusted odds ratio; ASPECTS, Alberta Stroke Program Early CT Score; CI, confidence interval; cOR, common odds ratio; mRS, modified Rankin Scale.

| **Analysis** | **Predictor** | **Ordinal mRS, cOR (95% CI)** | **p value** | **mRS 0-3, aOR (95% CI)** | **p value** |
| --- | --- | --- | --- | --- | --- |
| Model 2 (n=810) | ASPECTS 1-2 vs. 3-5 | 0.80 (0.53-1.23) | 0.311 | 0.89 (0.64-1.25) | 0.511 |
| Model 2 (n=810) | Successful reperfusion | 3.68 (2.31-5.85) | <0.001 | 4.10 (2.45-6.86) | <0.001 |
| Model 2 + number of maneuvers (n=768) | ASPECTS 1-2 vs. 3-5 | 0.83 (0.56-1.25) | 0.373 | 0.91 (0.68-1.22) | 0.527 |
| Model 2 + number of maneuvers (n=768) | Successful reperfusion | 3.64 (2.36-5.62) | <0.001 | 4.06 (2.85-5.80) | <0.001 |
| Model 2 + number of maneuvers (n=768) | Number of maneuvers | 0.90 (0.84-0.97) | 0.004 | 0.82 (0.71-0.94) | 0.005 |
| Model 2 + calendar year (n=810) | ASPECTS 1-2 vs. 3-5 | 0.80 (0.53-1.21) | 0.292 | 0.90 (0.64-1.26) | 0.524 |
| Model 2 + calendar year (n=810) | Successful reperfusion | 3.76 (2.41-5.86) | <0.001 | 4.36 (2.67-7.13) | <0.001 |
| Model 2 + calendar year (n=810) | Calendar year, per year | 0.95 (0.88-1.02) | 0.124 | 0.91 (0.85-0.98) | 0.014 |
| Model 2 + treatment era (n=810) | ASPECTS 1-2 vs. 3-5 | 0.81 (0.53-1.24) | 0.336 | 0.92 (0.65-1.30) | 0.632 |
| Model 2 + treatment era (n=810) | Successful reperfusion | 3.73 (2.37-5.86) | <0.001 | 4.28 (2.63-6.98) | <0.001 |
| Model 2 + treatment era (n=810) | Era 2020-2023 vs. 2015-2019 | 0.86 (0.68-1.10) | 0.224 | 0.72 (0.54-0.95) | 0.022 |
| Population-averaged model (n=810) | ASPECTS 1-2 vs. 3-5 | 0.83 (0.55-1.24) | 0.357 | 0.90 (0.65-1.25) | 0.517 |
| Population-averaged model (n=810) | Successful reperfusion | 3.79 (2.43-5.93) | <0.001 | 4.13 (2.52-6.76) | <0.001 |
| Onset- or last-seen-well-to-groin time (n=703) | ASPECTS 1-2 vs. 3-5 | 0.85 (0.52-1.37) | 0.495 | 0.89 (0.62-1.29) | 0.547 |
| Onset- or last-seen-well-to-groin time (n=703) | Successful reperfusion | 4.10 (2.65-6.36) | <0.001 | 4.61 (2.80-7.59) | <0.001 |

**Supplementary Table 10. Examination of the proportional-odds assumption.**
Separate logistic regression models were fitted at every dichotomization of the 90-day mRS using the covariates of Model 2, with standard errors clustered on center. Each row contrasts the better outcome category with the worse one; n better is the number of patients in the better category. Values are adjusted odds ratios (95% CI) for the better outcome, so that an odds ratio above 1 indicates a shift towards lower mRS scores. ^a^Corresponds to the acceptable-outcome models in Supplementary Table 5. ^b^Corresponds to the survival models in Supplementary Table 6. These threshold-specific estimates provide a descriptive assessment for two predictors in Model 2, not a formal test of proportional odds for all covariates. Estimates for the lowest mRS thresholds were imprecise because few patients reached these outcomes. ASPECTS indicates Alberta Stroke Program Early CT Score; CI, confidence interval; mRS, modified Rankin Scale; mTICI, modified Thrombolysis in Cerebral Infarction.

| **Outcome dichotomy** | **n better** | **ASPECTS 1-2 vs. 3-5** | **Successful reperfusion (mTICI 2b-3 vs. 0-2a)** |
| --- | --- | --- | --- |
| mRS 0 vs. 1-6 | 26 | 0.46 (0.03-6.11) | 3.55 (0.35-35.91) |
| mRS 0-1 vs. 2-6 | 79 | 1.14 (0.45-2.92) | 3.40 (1.28-9.04) |
| mRS 0-2 vs. 3-6 | 150 | 0.99 (0.40-2.46) | 3.78 (2.13-6.70) |
| mRS 0-3 vs. 4-6^a^ | 232 | 0.89 (0.64-1.25) | 4.10 (2.45-6.86) |
| mRS 0-4 vs. 5-6 | 343 | 0.66 (0.47-0.94) | 4.59 (2.74-7.69) |
| mRS 0-5 vs. 6^b^ | 449 | 0.86 (0.49-1.50) | 3.32 (1.98-5.58) |

**Supplementary Figure 1. Baseline ASPECTS composition of the analysis cohort by center and by year of admission.**
**A**, number of included patients per center, stacked by baseline ASPECTS. Centers are anonymised and ordered by the number of included patients; the label above each bar gives the percentage of that center’s patients with an ASPECTS of 1-2. Seven of the 23 centers contributed no patient with an ASPECTS of 1-2. **B**, the same composition by year of admission. The share of patients with an ASPECTS of 1-2 varied between 6.5% and 16.7% without a temporal trend. No patient with an ASPECTS of 0 was treated, so that value does not appear. ASPECTS indicates Alberta Stroke Program Early CT Score.

*Alternative text (accessibility): Two stacked bar charts. Panel A shows the number of included patients for each of 23 anonymised centers, stacked by baseline ASPECTS 1 to 5 and ordered by decreasing center volume, with the percentage of patients with ASPECTS 1-2 printed above each bar and ranging from 0 to 17 percent. Panel B shows the same composition for each year of admission from 2015 to 2023, with the ASPECTS 1-2 share ranging from 7 to 17 percent without a trend.*
